# Habitat fragmentation impact on insect diversity: opposing forces at patch and landscape levels

**DOI:** 10.1007/s10980-025-02133-w

**Published:** 2025-05-30

**Authors:** Antoine Perrin, Frank Rein, Philippe Christe, Jérôme Pellet

**Affiliations:** 1 Department of Ecology and Evolution, University of Lausanne, UNIL-Sorge, Biophore, CH-1015 Lausanne, Switzerland; 2 N+P Wildlife Ecology, Place St-François 6, CH-1003 Lausanne, Switzerland

**Keywords:** Lepidoptera, Orthoptera, Landscape changes, SLOSS, Grasslands

## Abstract

**Context:**

Habitat loss is widely recognized as a major threat to biodiversity, but the effects of habitat fragmentation, whether positive or negative, remain controversial. It has been suggested that these effects vary depending on the spatial scale studied (patch vs. landscape) and the biodiversity metric considered (α-, β-, or γ-diversity).

**Objectives:**

We aimed to test the contrasting effects of habitat fragmentation on insect diversity across different scales. Specifically, we tested whether habitat fragmentation negatively affect α-diversity at the patch scale, while having positive effects on β- and γ-diversity at the landscape scale.

**Methods:**

We conducted surveys of Lepidoptera and Orthoptera in 18 dry meadows of varying size and isolation in Switzerland. We assessed the effects of patch size and connectivity on species diversity (α-diversity), analyzed species turnover (β-diversity) between patches, and performed SLOSS analyses to compare cumulative species richness (γ-diversity) between patches.

**Results:**

Patch size and connectivity positively influenced α-diversity for both Lepidoptera and Orthoptera. However, at the landscape scale, multiple small patches supported equal or even higher γ-diversity than a single large patch of equivalent area. β-diversity increased with geographical distance between patches, indicating greater species turnover between more distant patches.

**Conclusion:**

Our results highlight that the effects of habitat fragmentation, whether positive or negative, are scale-dependent. While habitat fragmentation negatively affects α-diversity at the patch scale, it can enhance overall β- and γ-diversity at the landscape scale. These findings suggest that conservation strategies should consider both large and small habitat patches to maximize biodiversity.

**Supplementary Information:**

The online version contains supplementary material available at 10.1007/s10980-025-02133-w.

## Introduction

The importance of protecting biodiversity, both for the ecosystem services it provides and for its intrinsic value, has been widely recognised in recent decades (Millennium Ecosystem Assessment 2005). In this context, habitat loss and land-use change have been identified as the main drivers of biodiversity loss (Caro et al. 2022). Habitat loss often leads to fragmentation, where the remaining landscape habitat is divided into smaller and potentially more isolated patches (Fahrig 2003; Haddad et al. 2015; Hansen et al. 2020). Although there is a consensus in the literature that habitat loss has a significant negative impact on biodiversity (Pimm and Raven 2000; Fahrig 2003; Laurance 2010; Betts et al. 2017), the impact of habitat fragmentation remains a controversial issue that has generated intense and stimulating debate (reviewed in Miller-Rushing et al. 2019). Indeed, some studies argue that the effect of fragmentation on biodiversity is rather negative (Haddad et al. 2015; Fletcher et al. 2018), while others argue that it is rather positive (Fahrig 2017; Fahrig et al. 2019).

This debate is further complicated by the fact that the effects of fragmentation are not independent of habitat loss, as both processes may interact in ways that influence biodiversity patterns (Didham et al. 2012; Rybicki and Hanski 2013; Zhang et al. 2024). Additionally, habitat loss and fragmentation studies exhibit strong geographic and taxonomic biases, with nearly 85% conducted in North America and Europe, primarily focusing on temperate forests and birds (Fardila et al. 2017; Davison et al. 2021). Research has also primarily examined fragmentation at the patch level, focusing on habitat characteristics such as size and isolation (Fahrig 2003; Fardila et al. 2017), partly due to the strong initial influence of area and distance effects in island biogeography (MacArthur and Wilson 1967). This focus has led to the ongoing debate about the appropriate spatial scale at which to study habitat fragmentation (Fletcher et al. 2023a). Some studies argue that fragmentation should be examined at the landscape level only (e.g., increased patch number; Fahrig 2017; Fahrig et al. 2019), while others stress the importance of patch-level changes, such as increased edge effects, in shaping biodiversity responses (Fletcher et al. 2018).

Some authors have suggested that habitat fragmentation has a negligible effect on biodiversity compared to habitat loss. This idea was conceptualised in the Habitat Amount Hypothesis (Fahrig 2013), which suggests that the amount of habitat in a landscape is the main determinant of species richness, implying that the spatial configuration of patches in the landscape has no effect (Watling et al. 2020). Some studies have validated this hypothesis, while others have rejected it (reviewed in Martin 2018). This theory is currently the subject of much debate, particularly about what it does or does not imply about the effects of habitat fragmentation on biodiversity (Fahrig 2021; Saura 2021a, b). Saura (2021a) has shown that misinterpretation of the implications of this theory may be due to confusion over how to define habitat fragmentation (number, isolation, elongation or perforation of patches), the different spatial scales (patch, local landscape and region) and the different levels of biodiversity (α-, β- and γ-diversity) that we can study. This body of literature showed that both the spatial scale and the component of biodiversity considered determine whether the effects of habitat fragmentation are positive, negative or null.

Determining whether the effect of habitat fragmentation is positive or negative is crucial in conservation biology to answer the following question: Irrespective of the amount of habitat, is it more interesting to protect many small patches or a single large patch in a landscape (Fahrig et al. 2022)? One of the methods commonly used to test the positive or negative effect of fragmentation, independent of the amount of habitat at the landscape scale, is to compare species accumulation curves as a function of habitat area accumulation, starting from the smallest patch to the largest, or vice versa (hereafter referred to as the Single Large or Several Small (SLOSS) approach (Diamond 1975)). These comparisons typically suggest that a collection of small patches contains more species than a single large patch (Pellet et al. 2013; Fahrig 2020), likely due to higher landscape heterogeneity and increased opportunities for species with different habitat requirements (e.g., landscape complementation), supporting the positive effect on γ-diversity found at this scale (Fahrig 2017).

Here we wanted to test whether the conclusion regarding the effect of habitat fragmentation could lead to an apparently opposite result depending on the spatial scale used and the measure of biodiversity observed using an entomological dataset. This would indicate that we are using too general a term to define habitat fragmentation, which is a complex process (Riva et al. 2024). Specifically, the main objective of this study was to test whether there is a negative effect of habitat loss and fragmentation at the patch scale on α-diversity when we focus on patch size and isolation (as expected from all the works based on the theory of island biogeography; Haddad et al. 2015; Fletcher et al. 2018), while there is a positive effect of habitat fragmentation at the landscape scale using a SLOSS approach on γ-diversity (based on the results of the meta-analysis of Fahrig 2017).

To do this, we used an inventory of Lepidoptera and Orthoptera carried out in an isolated set of 18 mesophilic pastures and meadows of varying size and isolation. These taxa have been used for decades as indicators in open environments (Rákosy and Schmitt 2011; Solascasas et al. 2022; Chowdhury et al. 2023). Their larval diet, which is closely linked to specific host plants, their dependence on specific herbaceous structures and the presence of landscape structuring elements (such as bushes, hedgerows or forest edges) on a larger scale make them valuable indicators of the ecological quality of grasslands. Butterflies are relatively specialised and mobile organisms, whereas orthopterans are generalist herbivores with lower vagility. The study of these taxa provides a comprehensive perspective on how habitat and landscape configuration influence insect diversity and can help clarify how different measures of habitat fragmentation affect biodiversity patterns.

## Material and methods

### Data collection

**Fig. 1:**
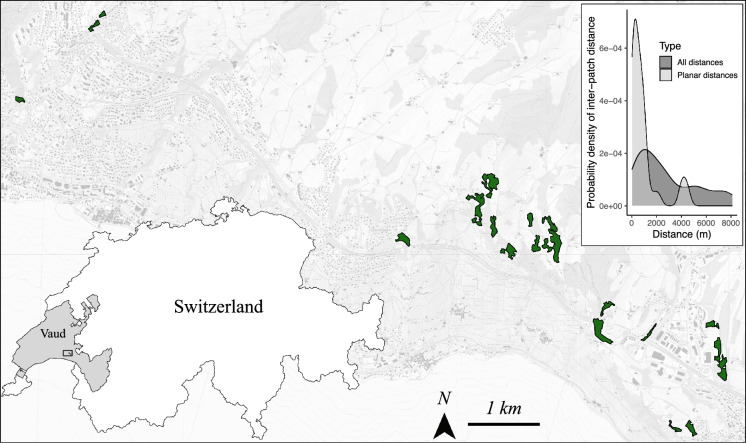
Location of the 18 studied mesophilic meadows and pastures (in green) in the canton of Vaud, Switzerland. The inset graph shows the density estimate of inter-patch distances calculated using the complete (dark grey) or planar (light grey) topologies. In the planar topology, only connections forming a minimal planar graph based on Voronoi polygons are considered, while in the complete topology, all possible connections between patches are included. The background map is provided by the Federal Office of Topography (swisstopo)

Insect surveys were carried out on 18 mesophilic meadows and pastures located at an altitude of 700 to 900 m above sea level on the northern shore of Lake Geneva, between the municipalities of Bourg-en-Lavaux and Chexbres, Switzerland (Eggenberg et al. 2001; Fig. 1). These south-facing sites range in size from 0.1 to 6.6 ha and consist of dry and nutrient-poor grasslands used either as hay meadows or pastures for cattle. Pastures are grazed, whereas meadows are mown. Eight sampling sites have mixed uses, alternatively being mown for fodder in summer and grazed in autumn. The surrounding matrix was composed of nutrient-rich grasslands (*Cynosurion* and *Arrhenatherion*) and forests (mostly *Fagenion*). As our study focused exclusively on nutrient-poor grasslands, patches were delineated based on their characteristic vegetation units, including *Mesobromion*, *Xerobromion*, *Geranion sanguinei*, and *Trifolion medii*. These patches are described in detail in Pellet et al. (2013).

Butterfly surveys were conducted in 2011, evenly spread between April and August, with an average of five visits per grassland per year, sufficient to detect most of the species present (Pellet 2023). During each visit, grasslands were sampled along a transect covering the diversity of vegetation units present. Visits lasted between 30 min and 2 h, depending on grassland size. Adult butterflies (including burnet moths but excluding other day-flying moths) were identified on sight and captured with a net when necessary. After handling and identification, all individuals were released. Orthoptera surveys were conducted between June and August 2014, with three survey sessions ensuring that the species accumulation curve approached saturation (Pellet 2023). Each visit lasted between 1 and 4 h, depending on grassland size. Surveys were conducted systematically, covering the entire grassland surface using a grid-like transect. All adult individuals were identified acoustically or by sight and were released after identification.

### Characteristics of grassland patches

We determined two common descriptors related to the size and isolation of each of the sampled patches (i.e. grasslands), which are assumed to reflect the structure of the patch resulting from habitat loss and fragmentation, respectively. We used the Graphab software (Foltête et al. 2021) to calculate patch area (‘surface’) and patch connectivity (‘connectivity’), measured using the 'flux metric' (F). F measures the area that a species can reach from the focal patch, considering the distance between patches. We set α values such that *p* = *e*^−α*dij*^ = 0.05 for a distance *d*_*ij*_ = 10′000 m. In other words, we set the probability of movement between two patches to 5% when the distance between them is 10′000 m. Although different butterfly and Orthoptera species exhibit varying degrees of vagility in the landscape, this threshold is relevant for the most vagrant species (Settele et al. 1999; Klaiber et al. 2017) and appropriately captures the spatial structure of our study area, where interpatch distances range from a few hundred meters to a maximum of 8 km (Fig. 1).

### Data analysis

#### Determinants of α species diversity

We used Generalized Linear Models, using the *stats* package of the R software, to determine the effect of the area and connectivity of grassland patches on species diversity, measured by the Shannon index, species richness and Pielou’s evenness of the Lepidoptera and Orthoptera communities (both of which being analysed separately) in the 18 sampled patches. These metrics were obtained using the R package *vegan*. Species evenness was obtained by dividing Shannon diversity by the logarithm of species richness. Models with the Shannon index or species evenness as the response variable included Gaussian-distributed errors, while the model with species richness as the response variable used Poisson-distributed errors. We assessed collinearity among area and connectivity variables using variance inflation factors (VIFs) with the vif function from the R package *car*. All VIFs were below 2, indicating no collinearity (Zuur et al. 2009). Model diagnostics were performed using graphical checks to assess residual homoscedasticity and normality. To account for the potential influence of the number of sampled individuals on observed species richness, we calculated the Chao1 estimator of total richness for each patch, using the R package *vegan*, for both Orthoptera and Lepidoptera, and used this metric as the response variable in the models. Additionally, we computed sampling completeness, defined as the ratio of observed richness to estimated total richness (Chao1), and tested whether patch area and connectivity were significantly related to sampling completeness.

#### Determinants of β species diversity

Between each pair of grassland patches, we calculated the Bray–Curtis dissimilarity index, using the R package *betapart*, and the geographic distance, using the Graphab software. To account for dependencies between pairwise distances, we fit linear models with maximum likelihood population effects (MLPE; Clarke et al. 2002). The covariate structure of the MLPE model includes a parameter that accounts for the correlation between distances involving the same sampling site. MLPE models were fitted using the gls function in the R package *nlme*, and correlation matrices were generated using the corMLPE function (https://github.com/nspope/corMLPE). The geographic distance was the explanatory variable and Bray–Curtis dissimilarity was the response variable.

#### Single large or several small analyses

We finally investigated the effects of grassland configuration at the regional scale on species richness by calculating cumulative species richness as a function of cumulative grassland area when progressively aggregating sampling sites in two different orders: (1) from the largest to the smallest patches and, (2) from the smallest to the largest patches (Quinn and Harrison 1988). We performed this analysis in R using the specaccum function from the *vegan* package with the “collector” method. Species occurrence data were first aggregated by site, and sites were then ordered based on their area. Cumulative species richness was computed at each step, summing the number of species as new patches were added. Simultaneously, we calculated the cumulative grassland area. This approach allows us to determine whether, given the same amount of habitat, many small patches support more or fewer species than a few large patches (Fahrig 2020).

## Results

A total of 2′043 individuals belonging to 52 Lepidoptera species and 2′814 individuals belonging to 26 Orthoptera species were identified. On average, a grassland contained 17 (SD = 5) Lepidoptera species and 9 (SD = 4) Orthoptera species. *Coenonympha pamphilus*, *Maniola jurtina*, *Melanargia galathea*, *Melitaea parthenoides*, *Polyommatus bellargus*, *Polyommatus icarus* and *Zygaena filipendulae* represented 68% of the total sampling of Lepidoptera individuals. *Platycleis albopunctata*, *Mecostethus parapleurus*, *Gomphocerippus rufus*, *Chorthippus biguttulus* and *Chorthippus parallelus* represented 71% of the total sampling of Orthoptera individuals (see Supplementary Material S1 for details).

At the patch level, both patch area and connectivity positively affect the α-diversity of Lepidoptera and Orthoptera communities. Specifically, Lepidoptera and Orthoptera species richness, as well as the Shannon index for Orthoptera, increases significantly with patch surface (Table 1). In addition, Lepidoptera species richness and the Shannon index for Orthoptera increases significantly with patch connectivity (Table 1). Other relationships are not significant (Table 1). We obtained identical results when using the Chao1 estimator instead of observed species richness, and there is no significant relationship between sampling completeness and patch surface or connectivity that could explain the significant results obtained for observed species richness (Table S1). Overall, these findings suggest that habitat loss and fragmentation at the patch scale negatively impact the α-diversity of both Lepidoptera and Orthoptera communities.

**Table 1:** Effect of the surface and connectivity of grassland patches on Lepidoptera and Orthoptera Shannon index, species richness and evenness

Response variable	Surface			Connectivity		
	Est (± SE)	z	p	Est (± SE)	z	p
Lepidoptera						
Shannon index	8.6e-06 (6.2e-06)	1.37	0.19	2.5e-06 (1.6e-06)	1.54	0.15
Richness	**1.4e-05 (5.4e-06)**	**2.62**	**0.009**	**4.6e-06 (1.8e-06)**	**2.55**	**0.01**
Evenness	− 1.3e-06 (1.7e-06)	− 0.78	0.45	− 5.4e-07 (4.4e-07)	− 1.22	0.24
Orthoptera						
Shannon index	**2.0e-05 (6.9e-06)**	**2.89**	**0.01**	**4.2e-06 (1.8e-06)**	**2.32**	**0.04**
Richness	**2.2e-05 (7.0e-06)**	**3.14**	**0.002**	2.5e-06 (2.4e-06)	1.04	0.30
Evenness	3.0e-07 (2.1e-06)	0.14	0.89	1.1e-06 (5.6e-07)	1.94	0.07

Significant relationships (p ≤ 0.05) are highlighted in bold

*Est* Standardised coefficient and *SE* Standard Error

**Fig. 2:**
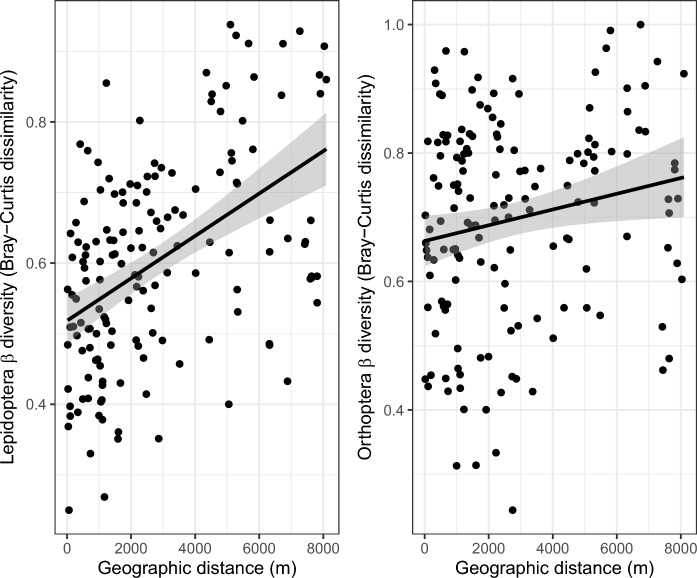
Dissimilarity in species composition (i.e., Bray–Curtis index) of Lepidoptera and Orthoptera communities between sampling patches as a function of geographic distance. Regression lines and their 95% confidence intervals are showed in dark and grey, respectively

The β-diversity of the two communities between patches are significatively explained by the geographical distance between them (Lepidoptera: t = 4.72, p < 0.0001; Orthoptera: t = 2.15; p = 0.034). The further apart two patches are, the greater the dissimilarity of both Lepidoptera and Orthoptera communities between the patches (Fig. 2).

**Fig. 3:**
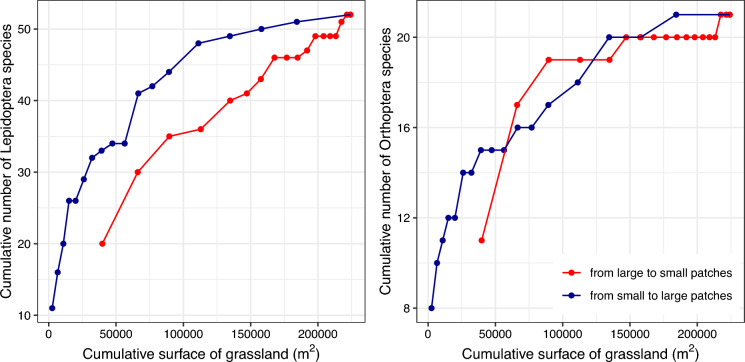
Cumulative number of Lepidoptera and Orthoptera species as a function of cumulative surface of grassland. Blue and red points indicate accumulation direction from smallest to largest patches or from largest to smallest, respectively

Although large patches support more species than small ones, at the landscape scale the cumulative species richness of several small patches is similar for the Orthoptera community and even higher for the Lepidoptera community than that of a single large patch of equivalent area (Fig. 3). This therefore suggest a positive effect of habitat fragmentation at the landscape scale on β- and γ-species diversities of both Lepidoptera and Orthoptera communities.

## Discussion

The results of this study highlight the complex and nuanced effects of habitat fragmentation on insect biodiversity, particularly within Lepidoptera and Orthoptera communities. Our results are consistent with many studies on this topic, which have shown that patch size and connectivity are critical determinants of species diversity (Collinge 2009; Haddad et al. 2015). The positive effects of these two metrics observed at the patch scale, where larger and more connected patches support higher species richness and diversity, underscore the importance of considering the spatial configuration of habitat patches when assessing the effects of fragmentation on biodiversity. Many studies have demonstrated that the detrimental effects of habitat fragmentation on local (α) diversity are due to increased extinction rates in smaller patches and reduced immigration in more isolated patches (MacArthur and Wilson 1967; Hanski and Ovaskainen 2000; Collinge 2009; Haddad et al. 2015).

Beyond these local effects, our β-diversity analyses reveal a pattern of decreasing species similarity with increasing distance between patches, consistent with the well-established concept of distance decay in biogeography and ecology (Nekola and White 1999; Soininen et al. 2007; Graco-Roza et al. 2022). This decline in similarity is primarily driven by dispersal limitation, as species are less likely to colonize distant patches. While environmental heterogeneity can also contribute to species turnover, its effect is likely less pronounced given the relatively small scale of our study area and relative homogeneity of habitat patches composed of similar vegetation associations. However, variation in land use and habitat management across patches may also play a role in shaping community composition. Grassland management, such as differences in grazing intensity and timing, mowing regimes, or landowner-specific practices, can influence species assemblages (Fiedler et al. 2017). For example, a single large patch might be uniformly mown or grazed, whereas a mosaic of smaller patches could support a diversity of management practices, leading to increased habitat heterogeneity and species turnover. Smaller, more fragmented patches may also be embedded in a structurally richer matrix with more hedgerows, woodland edges, or ecotones, which are known to support higher species richness by offering shelter, alternative resources, and connectivity for dispersing individuals (Ramírez-Delgado et al. 2022; Klimm et al. 2024). While our study does not explicitly quantify these structural elements, their potential contribution to observed β-diversity patterns warrants further investigation. In conclusion, although we lack genetic data to directly assess individual movements, the spatial patterns in species composition strongly suggest that both landscape connectivity and habitat heterogeneity play important roles in shaping biodiversity patterns in our study area. Interestingly, while fragmentation generally reduces local diversity, patch isolation also increases species turnover across the landscape, leading to higher regional (γ) diversity. This highlights the dual nature of fragmentation: it can be detrimental at the patch level but may promote species diversity at broader spatial scales (McGarigal and Cushman 2002; Fahrig 2003). These findings emphasize the need for conservation strategies that balance connectivity with habitat heterogeneity to maintain biodiversity in fragmented landscapes.

Our results add to the ongoing debate about the Habitat Amount Hypothesis, which posits that the total amount of habitat in a landscape is the primary determinant of species richness, with little or no effect of spatial configuration (Fahrig 2013). While our study supports the idea that large patches are critical for maintaining local diversity, it also highlights that small patches contribute significantly to γ-diversity. This is particularly evident in our finding that the cumulative species richness of multiple small patches can be equal to or greater than that of a single large patch of equivalent area, especially for Lepidoptera. These results suggest that small patches can serve as important refugia or stepping stones that enhance landscape connectivity and overall biodiversity (Fahrig 2020).

Our study highlights the need for a multi-scale and multi-faceted approach to conservation planning. The effects of habitat fragmentation on biodiversity cannot be fully understood without considering the spatial scale, the specific taxa involved and the aspect of biodiversity (α, β or γ) under investigation. This aligns with a broader pattern in the literature, which shows that habitat fragmentation alters both landscape structure and patch-level characteristics, with ecological responses often emerging from interactions across multiple spatial scales (Fletcher et al. 2023a, b). In fact, patch- and landscape-scale effects can operate independently, and extrapolating local responses to predict landscape-level outcomes can lead to incorrect predictions (Fahrig 2024). This complexity has major implications for conservation because neither patch-scale nor landscape-scale strategies alone can fully capture or mitigate the ecological consequences of habitat fragmentation. As Boyd et al. (2008) showed across thousands of threatened vertebrates, most species require conservation actions at multiple spatial scales. Conservation strategies should therefore be tailored to the specific context, considering the complex interactions between habitat size, connectivity and landscape heterogeneity. Furthermore, our results suggest that a combination of large, well-connected patches and smaller, isolated patches may be necessary to maximise biodiversity at both local and regional scales. On the other hand, restoring landscape-scale communities requires reducing the isolation of restored habitat patches to allow spontaneous recolonisation by species. Indeed, communities in restored (or new) habitat patches are driven solely by colonisation, whereas communities in existing isolated patches are driven by past populations (Watts and Hughes 2024). As the debate on habitat fragmentation continues, it is important to integrate these different perspectives to develop more effective conservation strategies that are sensitive to the complexity of ecological systems.

In conclusion, determining whether the effect of fragmentation on biodiversity is positive or negative is a difficult task and ultimately there is no single answer because the term biodiversity covers different aspects that do not respond in the same way. We therefore need to be careful about the clear conclusions of certain meta-analyses (e.g., Haddad et al. 2015; Fahrig 2017) when applying them in the field, and we need to be careful about which aspect of biodiversity is being studied to avoid implementing inappropriate measures to conserve biodiversity. Recent discussions on this topic and a joint publication by authors with differing views (Valente et al. 2023) point in the direction of a potential consensus for the future rather than continued disagreement, which will be beneficial for both landscape ecology and conservation biology.

## Supplementary Information

Below is the link to the electronic supplementary material.

Supplementary file1 (XLSX 17 KB)

Supplementary file2 (DOCX 15 KB)

## Data Availability

The data supporting the findings of this study are available within the article and its supplementary materials.
