# Supplementary material for: Habitat fragmentation impact on insect diversity: opposing forces at patch and landscape levels
Source: Landsc Ecol. 2025 May 30;40(6):113. doi: 10.1007/s10980-025-02133-w (PMC12122616; doi:10.1007/s10980-025-02133-w)
Supplement: Supplementary file 2 — Supplementary file2 (DOCX 15 KB) [file 10980_2025_2133_MOESM2_ESM.docx]

**Table S1: Effect of the surface and connectivity of grassland patches on Lepidoptera and Orthoptera species richness (Chao1 estimate) and sampling completedness.** Significant relationships (p ≤ 0.05) are highlighted in bold. Est = Standardised coefficient and SE = Standard Error.

|  |  | surface | | |  | connectivity | | |
| --- | --- | --- | --- | --- | --- | --- | --- | --- |
| response variable | | Est (±SE) | z/t | p |  | Est (±SE) | z/t | p |
| Lepidoptera | |  |  |  |  |  |  |  |
|  | Chao1 richness | **1.1e-05 (4.5e-06)** | **2.52** | **0.01** |  | **6.0e-06 (1.5e-06)** | **3.93** | **<0.0001** |
|  | sampling completeness | -4.4e-08 (3.8e-06) | -0.01 | 0.99 |  | -4.2e-07 (1.0e-06) | -0.42 | 0.68 |
| Orthoptera | |  |  |  |  |  |  |  |
|  | Chao1 richness | **2.3e-05 (6.7e-06)** | **3.48** | **0.0005** | | -2.7e-06 (2.2e-06) | -0.12 | 0.90 |
|  | sampling completeness | -1.2e-01 (2.2e-06) | -0.54 | 0.60 |  | **1.7e-06 (5.8e-07)** | **2.98** | **0.009** |
